# Predicting glioblastoma progression using MR diffusion tensor imaging: A systematic review

**DOI:** 10.1111/jon.13251

**Published:** 2024-12-09

**Authors:** Francesca M. Cozzi, Roxanne C. Mayrand, Yizhou Wan, Stephen J. Price

**Affiliations:** ^1^ Cambridge Brain Tumour Imaging Laboratory Division of Neurosurgery Department of Clinical Neurosciences Addenbrooke's Hospital University of Cambridge Cambridge UK

**Keywords:** diffusion tensor imaging, glioblastoma, recurrence, progression

## Abstract

**Background and purpose:**

Despite multimodal treatment of glioblastoma (GBM), recurrence beyond the initial tumor volume is inevitable. Moreover, conventional MRI has shortcomings that hinder the early detection of occult white matter tract infiltration by tumor, but diffusion tensor imaging (DTI) is a sensitive probe for assessing microstructural changes, facilitating the identification of progression before standard imaging. This sensitivity makes DTI a valuable tool for predicting recurrence. A systematic review was therefore conducted to investigate how DTI, in comparison to conventional MRI, can be used for predicting GBM progression.

**Methods:**

We queried three databases (PubMed, Web of Science, and Scopus) using the search terms: (diffusion tensor imaging OR DTI) AND (glioblastoma OR GBM) AND (recurrence OR progression). For included studies, data pertaining to the study type, number of GBM recurrence patients, treatment type(s), and DTI‐related metrics of recurrence were extracted.

**Results:**

In all, 16 studies were included, from which there were 394 patients in total. Six studies reported decreased fractional anisotropy in recurrence regions, and 2 studies described the utility of connectomics/tractography for predicting tumor migratory pathways to a site of recurrence. Three studies reported evidence of tumor progression using DTI before recurrence was visible on conventional imaging.

**Conclusions:**

These findings suggest that DTI metrics may be useful for guiding surgical and radiotherapy planning for GBM patients, and for informing long‐term surveillance. Understanding the current state of the literature pertaining to these metrics’ trends is crucial, particularly as DTI is increasingly used as a treatment‐guiding imaging modality.

## INTRODUCTION

Glioblastoma (GBM) is a fatally aggressive malignancy of the brain, with most patients rapidly succumbing to this tumor given its progressive and heterogeneous nature.^1^ Since 2005, the Stupp protocol has been pervasively used as the standard treatment regimen for GBM; subsequent to maximal safe surgical resection of the primary tumor, a patient receives concomitant radiotherapy and Temozolomide chemotherapy for 6 weeks, followed by maintenance chemotherapy for 6 months.^2^, ^3^, ^4^ Despite this, the median survival of these patients is less than 2 years.^1^ One of the critical reasons for this dismal prognosis is the inexorable progression of GBM following initial therapy.^4^ Unfortunately, recurrence (used interchangeably with progression herein) is not appreciable earlier on by conventional magnetic resonance imaging, which is the standard imaging modality used for diagnosis and surgical planning.

Importantly, although the following should be taken into consideration with the possible adverse effects from aggressive resection, there is a growing body of evidence (although based upon retrospective, non‐randomized data) demonstrating that the extent of tumor resection correlates positively with prolonged survival in GBM patients.^5^, ^6^, ^7^ Given the prognostic implications of extent of resection of GBM, the Response Assessment in Neuro‐Oncology (RANO) *resect* group has defined several categories for resection extent based upon contrast‐enhancing (CE) and non‐contrast‐enhancing (nCE) residual tumor observed on postoperative MRI.^7^ The RANO categories are separated into four main classes (Classes 1‐4), whereas Class 2 and Class 3 each have two sub‐classes (Class 2A, Class 2B, Class 3A, and Class 3B).^7^ Table 1 details the breakdown of each class with respect to CE and nCE post‐resection.^7^

**TABLE 1:** Response Assessment in Neuro‐Oncology *resect* group's categories for glioblastoma extent of resection.

Class		CE	nCE
1^α^		0	≤5 cm^3^
2^β^	2A^δ^	0	>5 cm^3^
	2B^ψ^	≤1 cm^3^	n/a
3^ω^	3A^ϕ^	≤5 cm^3^	n/a
	3B^γ^	>5 cm^3^	n/a
4^φ^		n/a	n/a

*Note*: Response Assessment in Neuro‐Oncology category names: ^α^“supramaximal CE resection”; ^β^“maximal CE resection”; ^δ^“complete CE resection”; ^ψ^“near total CE resection”; ^ω^“submaximal CE resection”; ^ϕ^“subtotal CE resection”; ^γ^“partial CE resection”; ^φ^“biopsy” with “no reduction of tumor volume”.^7^

Abbreviations: CE, contrast‐enhancing; cm^3^, cubed centimeters; n/a, not applicable to the respective class or subclass definition; nCE, non‐contrast‐enhancing.

Notably, even supramaximal CE resection does not infer total nCE resection, and so despite removal of all CE and potentially some removal of nCE, tumor inevitably recurs, and it typically recurs locally, relative to the initial tumor volume.^7^, ^8^, ^9^, ^10^, ^11^ It is well‐recognized that there is nCE tumor that contributes to recurrence, and this tumoral region can be appreciated on the T2‐weighted fluid‐attenuated inversion recovery (FLAIR) sequence.^8^, ^12^ In fact, biopsies of non‐enhancing regions in patients with GBM have been shown to contain the highest quantity of viable tumor cells compared to CE and necrotic regions.^13^ However, non‐enhancing regions comprise both tumor and edema and are typically only partially resected in a Class 1 “supramaximal CE resection” or Class 2A “complete CE resection,” given the apprehension to damage eloquent domain and unnecessarily remove regions that may be edema alone, but that cannot be distinguished from infiltrating tumor on FLAIR.^7^, ^12^, ^14^ As of 2023, when the European Society for Radiotherapy and Oncology–European Association of Neuro‐Oncology guideline on radiotherapy targets for GBM was published, no consensus regarding the radiotherapy treatment margin for the T2/FLAIR volume had been reached, given the limited available evidence and difficulty in distinguishing non‐enhancing infiltrative tumor from edema on this imaging sequence.^14^

Consequently, conventional MRI has shortcomings that obscure the early identification of infiltrating tumor, whereas imaging modalities that are more sensitive to microstructural architecture and occult tumor progression are needed to improve treatment potency and precision. In contrast to conventional MRI, diffusion tensor imaging (DTI) has been shown to detect occult white matter tract infiltration and tumor progression at earlier timepoints compared to the former.^15^, ^16^, ^17^ DTI is a modified technique of diffusion‐weighted imaging that is sensitive to anisotropic diffusion, defined as the directional diffusion of water molecules preferentially along axonal fibers, or white matter tracts.^18^ When the diffusion of water molecules is captured by diffusion MRI acquisition, the molecules have only had time to diffuse just tens of micrometers, which is why this imaging modality is advantageous for probing the local microenvironment.^19^ The sensitivity of DTI for assessing microstructural integrity in vivo is invaluable, as it can reveal subtle alterations to white matter tracts in a variety of disease states, including brain tumors.^18^

In fact, a study by van den Elshout et al. reported that in a 14‐day timeframe between preoperative MR scans for a population of 78 GBM patients, tumor growth was found to be significantly more frequent in the parallel, colinear orientation to white matter tracts compared to the perpendicular orientation.^20^ This is key, for knowing that tumor cells infiltrate surrounding tissue by migrating along these tracts (in a related manner to how water molecules diffuse along these tracts) may help to refine treatment approaches.^17^ The point of intrigue is to capture—or predict—the occult infiltration of white matter as early as possible, so as to inform primary treatment planning, preferentially, or salvage treatment.

The aim of this study was thus to conduct a systematic review, in adherence to the Preferred Reporting Items for Systematic reviews and Meta‐Analyses (PRISMA) 2020 guidelines, to investigate how DTI can be used to detect GBM recurrence, with emphasis on DTI metrics and features that have been associated with this tumor's progression. Amongst these are DTI‐*p* and DTI‐*q*, which are isotropic and anisotropic components, respectively, of the decomposed diffusion tensor; this “p:q decomposition” is a technique described by Peña et al.^21^ Two other metrics that are most commonly derived from DTI's tensor model are mean diffusivity (MD) and fractional anisotropy (FA). MD is a quantity that speaks to the diffusion average amongst every direction; to note, a region with unimpeded diffusion would have a relatively high MD, whereas the MD would be relatively low in areas where diffusion is more incumbered.^22^ On the other hand, FA is a measure of the diffusion variation amongst different directions, ranging in value from 0 (mainly isotropic) to 1 (mainly anisotropic).^22^ FA is low (closer to 0) in areas where diffusion is unimpeded, and it is high (closer to 1) in areas where diffusion is restricted in a directionally‐dependent way.^22^ Relative values for the two most common metrics (MD and FA) in different biological landscapes within the brain are summarized in Table 2.^22^

**TABLE 2:** Common diffusion tensor imaging metrics in different biological landscapes within the brain.

	White matter^a^	White matter^b^	Gray matter	CSF
Mean diffusivity	Moderate	Moderate to low	Moderate to low	High
Fractional anisotropy	High	High to moderate	Low	Low

^a^ White matter containing a single fiber.

^b^ White matter containing crossing fibers.

Moreover, two additional DTI‐derived scalar quantities that may be measured are axial and radial diffusivity; the former pertains to diffusion along the principal diffusion direction (ie, the axis aligned with a fiber bundle's direction), whereas the latter pertains to diffusion perpendicular to the axis of the principal diffusion direction.^22^ Metrics presented in the “Results” section will either be standard scalar parameters of DTI (ie, FA and MD) or metrics derived from more advanced, higher order DTI models, such as free‐water‐corrected (FWC) parameters.^23^ The purpose of investigating these metrics was to consolidate reported patterns in the literature to date and to offer a comprehensive understanding of similarities and differences across multiple studies. Knowledge of these patterns may help to predict progression and recurrence of GBM earlier on in the disease course, which may impact both initial treatment planning (ie, extent of resection and radiotherapy target volumes) and salvage treatment regimens.

Finally, there are instances when recurrence lies beyond the local CE region and radiotherapy treatment margin, and occult infiltration along white matter tracts in distant regions (ie, the contralateral hemisphere) is initially undetectable on standard imaging.^4^, ^17^, ^24^ The use of DTI to predict both distant progression and progression at all is important, and understanding the ways in which it has already been reported to distinguish recurrence may prove helpful in that endeavor. Although there have been reviews on the use of DTI metrics to predict overall survival in glioma patients, we asked a different question for a specific patient population: that is, how can DTI, compared to conventional MR imaging, be used to predict recurrence in GBM patients in order to detect progression earlier in time?

## METHODS

### Search strategy and information sources

PubMed, Web of Science, and Scopus were searched in April 2024 with the following search terms: (diffusion tensor imaging OR DTI) AND (glioblastoma OR GBM) AND (recurrence OR progression).

### Inclusion criteria

The results of the initial search were systematically reviewed with the following inclusion criteria:

1. Original articles (excluding reviews of any kind)
2. Written in the English language
3. Reported data specific to GBM patients
4. Reported on the primary endpoint of DTI‐related data for characterizing and/or predicting GBM progression following initial treatment

### Exclusion criteria

Articles were excluded for the following reasons:

1. Focused on other endpoints without detailing how DTI metrics can be used to predict/characterize progression

Of note, survival endpoints were not included in the focus here, because a systematic review on the use of diffusion MRI metrics specifically for predicting survival outcomes in GBM patients was published in 2020 by Brancato et al.^25^

### Selection process

Inclusion and exclusion criteria were applied systematically, as all populated articles were reviewed. Rayyan was used to screen automatically for duplicate records.^26^

### Data collection process and data items

Data were systematically extracted from the articles and included: type of study; number of GBM patients with recurrence/progression; treatment prior to recurrence/progression; and outcomes pertaining to DTI‐related metrics and patterns.

### Synthesis methods and effect measures

All included studies contained information on the aforementioned data items and were therefore eligible for synthesis and presentation within the “Results” section. Summary tables were tabulated from the extracted data and are presented in the “Results” section.

### Risk of bias assessment

Risk of bias was assessed for the included studies, and findings as to the overall quality of the included articles and their associated risk of bias are reported in the “Results” section. Level of evidence for each article was assessed and determined using the Oxford Centre for Evidence‐Based Medicine Levels of Evidence Working Group's “Levels of Evidence Table” as a reference guide.^27^ When assessing each article, reference was made to the table's diagnostic question row, which aligned most closely with the question posed in this article, pertaining to the utility of DTI (a diagnostic imaging instrument) for predicting/characterizing GBM recurrence.

## RESULTS

### General findings

**FIGURE 1:**
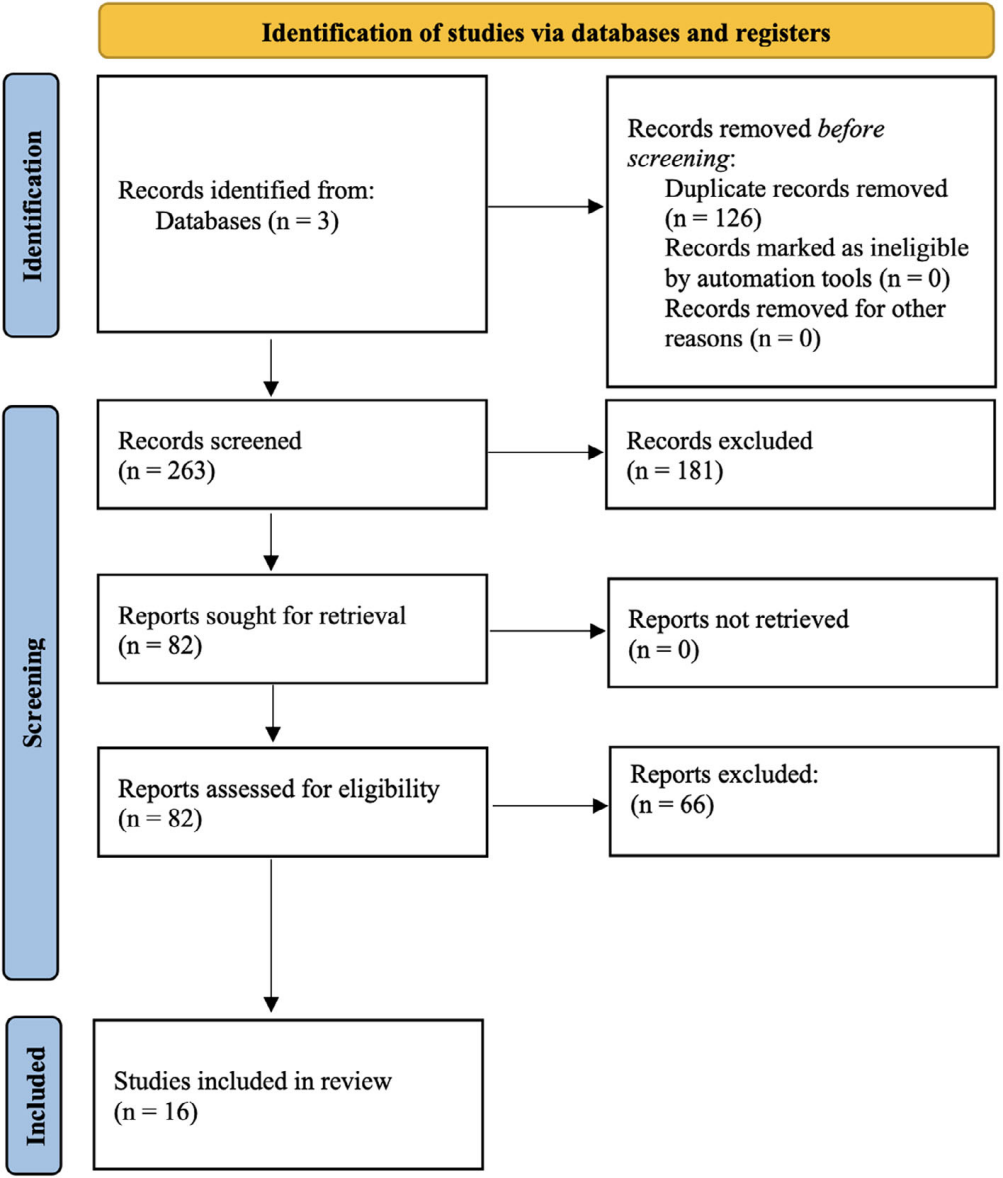
Preferred Reporting Items for Systematic reviews and Meta‐Analyses flow chart of the sequence of steps taken in this systematic review to ascertain articles meeting inclusion criteria. *Note: n*, number.

The search yielded 389 total results, of which 16 studies met inclusion criteria. The sequential process of this systematic review is presented in the PRISMA (2020) flow diagram in Figure 1.^28^ From these 16 studies, there was a total of 394 patients with reported GBM recurrence. Of what was reported: 42 patients underwent surgery alone; 228 patients underwent surgery and chemoradiotherapy; 26 patients had surgery and radiotherapy; and 36 patients had radiotherapy alone. In 2 studies, patients underwent surgery in addition to a single or a combination of adjuncts or supportive care, but the adjunct/supportive care was unspecified for the recurrence patients specifically; these patients totaled 62 across the 2 studies. Moreover, 6 of the included studies were prospective, and 10 were retrospective. The studies were published between 2004 and 2023 (inclusive), most (*n* = 9, or 56%) of which were published just within the last 5 years (Table 3). The following sections address some of the most commonly described DTI‐related metrics (summarized in Table 4) and associated tractography/connectomics, with comprehensive findings for all included articles detailed in Table 5.

**TABLE 3:** Summary characteristics of included articles.

Author	Study type	Level of evidence^a^	*N* Recurrence patients	Treatment pre‐recurrence
Wei et al.^17^	Prospective	4	2	Surgery
Feng et al.^33^	Prospective	4	31	Surgery CRTx
Yan et al.^32^	Retrospective	4	37 (Training) 20 (Test)	Surgery CRTx + Adjuvant TMZ
Metz et al.^30^	Prospective	4	35	Surgery
Jin et al.^15^	Retrospective	4	30	RTx
Witulla et al.^39^	Retrospective	4	7	Surgery RTx
Kim et al.^35^	Retrospective	4	59 (Training) 24 (Test)	Surgery CRTx
Li et al.^36^	Prospective	4	57 (115 in Total Cohort)	Surgery + CRTx + adjuvant TMZ (*N* = 84/115) Surgery + RTx (*N* = 20/115) Surgery + supportive care (*N* = 11/115)
Peeken et al.^41^	Retrospective	4	14	Surgery RTx
Wang et al.^34^	Retrospective	4	21	Surgery CRTx
Stecco et al.^31^	Prospective	4	17	Surgery CRTx
Khayal et al.^29^	Prospective	4	19	Surgery CRTx PKC‐β inhibitor
Li et al.^40^	Retrospective	4	5	Surgery + CTx and/or RTx
Krishnan et al.^38^	Retrospective	4	6	Stereotactic RTx
Price et al.^37^	Retrospective	4	9	Surgery + RTx (*N* = 4) Surgery/biopsy + no adjunct (*N* = 5)
Price et al.^16^	Retrospective	4	1	Surgery RTx

Abbreviations: CRTx, chemoradiotherapy; CTx, chemotherapy; *N*, number; PKC, protein kinase C; RTx, radiotherapy; TMZ, Temozolomide.

^a^ The level of evidence was determined using the Oxford Centre for Evidence‐Based Medicine Levels of Evidence Working Group's “Levels of Evidence Table,” with use of the table's diagnostic question row. This row was most related to the question posed in this article, regarding the utility of diffusion tensor imaging, a diagnostic imaging instrument, for predicting/characterizing glioblastoma recurrence.

**TABLE 4:** Summary of common diffusion tensor imaging related metrics reported amongst included articles.

Author	Metrics					
	MD	FWC‐MD	FA	FWC‐FA	p	q
Feng et al.^33^			↑^a^			
Yan et al.^32^			↓		↓	ND
Metz et al.^30^	↓	↓	↓	↓		
Jin et al.^15^	↑^b^		↓^b^			
Li et al.^36^					↓^c^	↑^c^
Wang et al.^34^	ND^a^		↑^a^			
Stecco et al.^31^			↓			
Khayal et al.^29^			↓			
Price et al.^16^			↓^d^		↑	↓

Abbreviations: FA, fractional anisotropy; FWC, free‐water‐corrected; MD, mean diffusivity; ND, no difference or variation between progression versus non‐progression; *p*, DTI‐*p* isotropic metric; *q*, DTI‐*q* anisotropic metric.

^a^ Recurrence compared to radiation necrosis or pseudoprogression.

^b^ Comparison of metrics at time of recurrence to other timepoints prior to recurrence.

^c^ In non‐enhancing region, positively associated with rate of progression.

^d^ In peritumoral area.

**TABLE 5:** Detailed outcomes across all included studies.

Author	Standard DTI metrics	Higher order DTI metrics and tractography/connectomics
Wei et al.^17^	n/a	Furthest distance of recurrence from tumor centroid positively correlated with disruption of distant region connectome (*p* < .001) Distant recurrence was identified on follow‐up scans in 2 cases, whereas preoperative MRI did not reveal the occult tumor infiltration of these distant regions Distant lesions in both cases traced through white matter connections to the primary lesion Preoperative T1c images did not show any lesion at the location of recurrence
Feng et al.^33^	FA values for tumor recurrence significantly higher than radiation necrosis (*p* = .001) Axial diffusion coefficient and radial diffusion coefficient values significantly lower for tumor recurrence vs. radiation necrosis (*p* = .003 for both) FA best distinguished tumor recurrence vs. radiation necrosis: AUC, 0.798; sensitivity, 80.6%; specificity, 66.6%	n/a
Yan et al.^32^	FA lower in region of progression vs. 5 mm of non‐progression (*p* = .041) Significant decrease in DTI‐*p* component in region of progression vs. 5 and 10 mm of non‐progression (*p* < .001) In progression regions, DTI‐*q* did not demonstrate variation between progression and non‐progression rCBV higher in regions of progression vs. 15‐20 mm of non‐progression	n/a
Metz et al.^30^	n/a	Compared to non‐corrected maps, there was a significantly higher AUC in a generalized mixed‐effect model using FWC‐FA maps (*p* < .001) FA values were significantly lower for areas that eventually contained tumor recurrence vs. areas of wholly recurrence‐free edema In 3 percentiles (10th, 50th, and 90th) of tissue‐volume‐fraction values, there were significant differences in FWC‐FA maps in regions of recurrence vs. recurrence‐free edema (*p* = .00112, .00314, and .00007, respectively) Non‐corrected FA maps demonstrated significant differences between the 2 regions in the 90th percentile only (*p* = .0003) For MD values after FWC, only the 90th percentile showed a significant difference between regions with edema and later recurrence vs. regions with pure edema, with regions of eventual recurrence having lower MD (*p* = .04648400)
Jin et al.^15^	At recurrence, mean FA significantly lower than: 1 mo post‐RTx, 4 mo pre‐recurrence, and 2 mo pre‐recurrence (*p* < .05), with a decrease of 30.8%, 22.9%, and 19.2% from 1 mo post‐RTx, to 4 mo pre‐recurrence, and to 2 mo pre‐recurrence, respectively Over time, MD, axial diffusivity, and radial diffusivity values showed an increasing trend FA images demonstrated white matter degeneration at area of recurrent tumor at 4 and 2 mo pre‐recurrence, whereas tumor was not visible at these pre‐recurrence timepoints on T1WI	n/a
Witulla et al.^39^	n/a	In 1/7 patients, fiber tracking showed clear connection from primary tumor to distant recurrence 6/7 patients with distant recurrence showed weak connections not usable for defining the RTx planning target volume
Kim et al.^35^	Training set: For the prediction of 6‐mo progression, FA + nCBV had the best performance (sensitivity, 94.1%; specificity, 57.1%); better than FA or nCBV alone Test set: FA + nCBV similarly yielded the best performance (sensitivity, 80.0%; specificity, 63.2%), compared to FA or nCBV alone	n/a
Li et al.^36^	Decreased DTI‐*p* and increased DTI‐*q* components in non‐enhancing area was significantly and positively associated with rate of progression (*p* = .010) Increased DTI‐*p* and decreased DTI‐*q* components in non‐enhancing area was negatively associated with rate of progression (*p* = .040)	n/a
Peeken et al.^41^	n/a	In 13/14 patients, volumes containing recurrent tumor overlapped with infiltrative gross tumor volumes (defined using tissue volume and FA maps that were FWC)
Wang et al.^34^	For tumor progression compared to pseudoprogression: No significant difference in median MD (*p* > .05) Significantly higher rCBV_max_ (*p* = .007) Significantly higher FA (*p* = .008) Significantly higher linear anisotropy (*p* = .04) Significantly higher planar anisotropy (*p* = .002) Significantly decreased spheric anisotropy (*p* = .004)	n/a
Stecco et al.^31^	Over time, low FA in enhancing regions did not change; however, FA in hyperintense perilesional tissue showed a significant decrease from post‐op/pre‐RTx to time of tumor progression	n/a
Khayal et al.^29^	For progressors vs. non‐progressors, no significant difference in median nFA at pre‐, mid‐, or post‐RTx in contrast‐enhancing lesion, non‐enhancing lesion, and T2 hyperintense regions From mid‐ to post‐RTx within both contrast‐enhancing and non‐enhancing lesion areas, significant percent change in nFA (*p* = .0396 and .0421, respectively), with a greater percent decrease observed in progressors compared to non‐progressors (−13% and −9% vs. −5% and −2%, respectively) From mid‐ to post‐RTx, significant median nFA change in the contrast‐enhancing lesion in progressors (*p* = .001); no observed significant differences in the non‐enhancing and T2 hyperintense lesions Mid‐RTx contrast‐enhancing lesion normalized eigenvalues 1 and 2 significantly higher in non‐progressors vs. progressors (not seen in non‐enhancing lesion and T2 hyperintense lesion)	n/a
Li et al.^40^	Diffusion time for peri‐tumoral volume of interest was higher than diffusion time in region of tumor recurrence, suggesting that tumor is inclined to grow in a faster diffusion area (ie, along white matter tracts) GBM recurrence patterns and DTI diffusion patterns were shown to be correlated	n/a
Krishnan et al.^38^	n/a	In 4 of these patients, paths of diffusion identified from the primary tumor site to the secondary site of progression In one of these patients, reconstructed diffusion path from primary tumor location predicted location of spread/progression (tractography useful for predicting tumor migration) Results support that tumor cells migrate in the direction along DTI pathways
Price et al.^37^	3/9 had diffuse recurrence, with global increase in tumor size (diffuse pattern defined as when the abnormality of DTI‐*p* exceeded DTI‐*q* in all directions) 6/9 had local recurrence, with tumor progression in the direction along which the abnormality of DTI‐*p* exceeded that of DTI‐*q*	n/a
Price et al.^16^	Initial primary tumor identified in right frontal lobe, with recurrent tumor 9‐mo post‐resection appearing in the contralateral left frontal lobe At time of recurrence, no apparent tumor infiltration of corpus callosum on T1WI and T2WI, but FA maps at this timepoint did show effects in the corpus callosum, specifically the genu (predominant at higher slices) Decrease in FA in peritumoral area Compared to controls, patient had low DTI‐*q* values (*p* = .03) and increased DTI‐*p* values (*p* = .05) in higher slices of genu of corpus callosum In tumor‐infiltrated regions, DTI‐*p* was increased DTI abnormalities in corpus callosum appeared 6 weeks prior to recurrent tumor in that region becoming visible on CT scan	n/a

Abbreviations: AUC, area under the curve; DTI, diffusion tensor imaging; DTI‐*p*, isotropic DTI component; DTI‐*q*, anisotropic DTI component; FA, fractional anisotropy; FWC, free water correction; GBM, glioblastoma; MD, mean diffusivity; mm, millimeter(s); mo, month(s); n/a, not applicable; nCBV, normalized cerebral blood volume; nFA, normalized FA; rCBV, relative cerebral blood volume; RTx, radiotherapy; T1c, T1‐weighted contrast; T1WI, T1‐weighted imaging; T2WI, T2‐weighted imaging; vs., versus.

### Fractional anisotropy

Several DTI parameters were reported across the included studies. With respect to FA in particular, 6 studies reported decreased FA values in areas of recurrence and/or for progressors compared to non‐progressors (Table 4). Specifically, there were reports of: significantly lower mean FA at recurrence compared to pre‐recurrence (all post‐radiotherapy)^15^; significant percent change in normalized FA from mid‐ to post‐radiotherapy, with a larger percent decrease in progressors versus non‐progressors^29^; significantly lower FWC FA in regions with eventual recurrence compared to non‐recurrence regions^30^; decreased FA in peritumoral areas^16^; significantly decreased FA values in perilesional tissue from pre‐radiotherapy to the time of tumor progression^31^; and FA values lower in an area of progression compared to non‐progression.^32^ Additionally, Feng et al. reported FA values being higher in regions of tumor recurrence compared to radiation necrosis, and Wang et al. reported significantly higher FA values in areas of true tumor progression versus pseudoprogression.^33^, ^34^ Kim et al. found that when FA and cerebral blood volume (CBV) radiomics for peritumoral regions were combined, this joint model demonstrated a better predictive value for local progression compared to FA or CBV alone.^35^

### DTI‐*p* and DTI‐*q* abnormalities

Amongst the included studies, there was some variability in reports on DTI‐*p* and DTI‐*q* components for recurrence (Table 4). Li et al. reported that decreased *p* and increased *q* in the non‐enhancing region correlated positively and significantly with the rate of tumor progression, whereas an increased *p* and decreased *q* in the non‐enhancing region correlated negatively with the rate of progression.^36^ Similarly, Yan et al. reported that in areas of progression, *p* was significantly decreased compared to non‐progression regions (and *q* did not demonstrate a difference between progression and non‐progression regions).^32^ Another study reported that a case of GBM recurrence, compared to controls, had low *q* values and increased *p* values for recurrence, with the *p* component increased in tumor‐infiltrated regions.^16^ Price et al. additionally defined patterns of invasion, with local recurrence defined as when tumor progression occurs along one direction in which the abnormality of *p* exceeds that of *q*, and diffuse recurrence when *p* exceeds *q* in all directions with a global increase in tumor size.^37^

### Connectomics, tractography, and other DTI metrics

Three studies reported white matter degeneration visible on DTI before recurrence became apparent on conventional imaging.^15^, ^16^, ^17^ Price et al. reported DTI abnormalities at a site of recurrence 6 weeks before recurrent tumor in the same location became visible on a CT scan.^16^ Krishnan et al. reported diffusion paths from the primary tumor site to the site of progression.^38^ In 1 patient from this study, when a path was reconstructed from the primary tumor, the path predicted the tumor's direction of spread.^38^ Similarly, Wei et al. “found that the higher distant region [connectome] disruption was positively correlated with the furthest recurrence distance from tumor centroid,” with two cases in which “the disrupted distant regions indicated occult tumor invasion invisible on the preoperative MRI.”^17^ One study was more doubtful about the utility of DTI and tractography, finding only 1 of 7 patients to have fiber tracking that delineated a clear connection between primary and recurrent tumor that was able to be used for setting the planning target volume for radiotherapy.^39^ Another study showed tumor to definitively grow in regions with faster diffusion (ie, along white matter tracts), as diffusion time around tumor was reported to be higher in the region of recurrence, with GBM recurrence and DTI diffusion patterns correlating.^40^ One other report defined a new volume area based upon tissue volume and FWC‐FA maps, where recurrent tumor was shown to intersect with this newly defined region.^41^

Other DTI metrics that were reported amongst the 16 studies included axial diffusion coefficient, radial diffusion coefficient, and MD. Compared to radiation necrosis, axial diffusion coefficient and radial diffusion coefficient values were reported to be significantly lower in regions of tumor recurrence.^33^ Tumor progression, compared to pseudoprogression, has also been shown to have significantly higher linear anisotropy and planar anisotropy, with significantly decreased spheric anisotropy and no significant difference in median MD.^34^ Another study reported that MD values with free water correction demonstrated a significant difference between regions with eventual recurrence and regions of pure edema at the 90th percentile, with lower MD values in regions of later recurrence.^30^ Two studies showed regions of tumor progression to have higher relative CBV compared to pseudoprogression or non‐progression regions.^32^, ^34^

### Risk of bias in included studies

The overall quality of the 16 included studies was poor, with risk of bias stemming from single‐center populations from which data were predominantly collected. Additionally, in most of the included studies, sample size was relatively small. Retrospective study designs and small cohort sizes limited the generalizability of results and the statistical power of some of the reported data.^35^ Time constraints and costs were also reported as possible limitations on data acquisition and results.^33^ Moreover, there were some technical inconsistencies, including the acquisition of MR imaging at different magnetic strengths.^15^

## DISCUSSION

The findings in this systematic review elucidate how DTI may be used for identifying, describing, and predicting progression patterns of GBM, with metrics that could be considered when planning surgical and radiotherapy treatment regimens.^15^, ^16^, ^17^, ^30^, ^32^, ^34^, ^35^, ^36^, ^37^, ^38^, ^39^, ^40^, ^41^ Although the overall quality of evidence amongst the included studies is low, the several patterns and trends identified in this review speak to the potential utility of DTI metrics (ie, MD, FA, *p*, and *q*) and associated tractography/connectomics for elucidating occult tumor infiltration beyond the CE primary lesion. Some metrics have been shown to offer insight into the path to recurrence before progression is visible on conventional imaging. Nonetheless, in order to effectively discuss these metrics and findings in a digestible way, it is first important to understand the fundamentals of DTI and how these metrics are derived.

To start, DTI is an advanced magnetic resonance imaging modality coupled with the addition of magnetic field gradients for rendering an image that captures the diffusion of water molecules in one direction; this technique is then applied in many directions for engendering a 3‐dimensional tensor model.^42^ The tensor is a symmetric, 3 × 3 matrix with components that represent 3‐dimensional displacements.^43^ This model assumes a Gaussian distribution of diffusion per voxel.^43^ In addition to a matrix, which will be summarized shortly, the tensor model can be visualized as an ellipsoid, particularly when describing diffusion in an anisotropic medium.^43^ In such a medium, there is one principal direction along which the diffusion of water molecules is fastest, and the elongated axis of the diffusion ellipsoid indicates this preferred direction, from which white matter tract orientations can also be deduced.^42^, ^43^ From a mathematical perspective, the tensor takes the following matrix form:^21^

$$D=\left[\begin{array}{ccc} D_{xx} & D_{xy} & D_{xz}\\ D_{yx} & D_{yy} & D_{yz}\\ D_{zx} & D_{zy} & D_{zz} \end{array}\right]$$

Of these 9 components within the matrix, there are 6 independent components of the tensor, because of the symmetrical components on either side of the tensor's principal diagonal (ie, *D_xz_* = *D_zx_*).^21^ In other words, there are 6 off‐diagonal components altogether, but of these are 3 symmetrical pairs. Moreover, the 3 left‐to‐right diagonal components in the matrix above (*D_xx_*, *D_yy_*, and *D_zz_*) highlight the diffusion coefficients along the primary axes (*x*, *y*, and *z*), whereas the 3 off‐diagonal components (*D_xy_*, *D_xz_*, and *D_yz_*) represent the covariance of diffusion between two perpendicular axes (ie, *x* and *y* for *D_xy_*).^21^, ^43^

When diffusion is only along the primary axes, however, these off‐diagonal components are equal to zero (given zero correlation with respect to diffusion between the axes), and the only nonzero components are those lying along the primary axes themselves (the 3 left‐to‐right diagonal components in the aforementioned matrix).^43^ In this instance, there are 3 eigenvalues that represent diffusivity along each of the primary axes, whereas the 3 eigenvectors represent the orientation of each of these primary axes.^43^ Additionally, the eigenvector corresponding to the largest eigenvalue represents the principal (preferred) direction of diffusion, meaning that in a voxel, the tensor orientation given by the principal eigenvector is taken to lie in parallel to the predominant fiber orientation.^43^ Overall, from these values, several common metrics can be derived.

Regarding the several metrics reported amongst the 16 included articles in this systematic review, 6 studies reported that FA values are lower in areas of recurrence.^15^, ^16^, ^29^, ^30^, ^31^, ^32^ FA is a DTI measure with high sensitivity for microstructural changes.^44^ It is defined by the formula:

$$\mathrm{FA}=\sqrt{\frac{3}{2}}\frac{\sqrt{{\left(\lambda_{1}-\mathrm{MD}\right)}^{2}+{\left(\lambda_{2}-\mathrm{MD}\right)}^{2}+{\left(\lambda_{3}-\mathrm{MD}\right)}^{2}}}{\sqrt{\lambda_{1}^{2}+\lambda_{2}^{2}+\lambda_{3}^{2}}},$$

where MD is the mean diffusivity, and *λ*_1_, *λ*_2_, and *λ*_3_ are the 3 eigenvalues of the diffusion tensor.^21^ MD is calculated as the quotient of the trace (sum of the three eigenvalues) divided by 3, as follows:^21^

$$\mathrm{MD}\;=\frac{\lambda_{1}+\lambda_{2}+\lambda_{3}}{3}$$

Like MD, FA is a scalar. Therefore, FA does not describe the shape/distribution of the tensor, and it is not highly specific to the type of microstructural change that is occurring: that is, axial or radial.^44^ Nonetheless, amongst the several DTI scalars available, FA is the most commonly used, and its utility stems from its elevated sensitivity to changes in axonal fiber microstructure.^45^ Although FA is limited in its specificity for discerning what kind of change is occurring amongst pathological states, its high sensitivity to change in and of itself offers valuable information to the observer.^44^, ^45^

Interestingly, Jin et al. showed that the mean FA value at the timepoint of recurrence was significantly lower than the mean FA values at three timepoints beforehand: that is, 1 month post‐radiotherapy, 4 months pre‐recurrence, and 2 months pre‐recurrence.^15^ Additionally, the mean FA value 2 months pre‐recurrence, compared to 1 month post‐radiotherapy, was significantly lower by 11.2%; moreover, white matter deterioration was apparent on FA images at both 4 and 2 months pre‐MR‐visible recurrence.^15^ Moreover, recurrence occurred at the intersection of the internal capsule and geniculocalcarine tract, both of which are predominant white matter tracts.^15^ This speaks to the utility of FA for identifying recurrence before it is appreciable on conventional MR imaging.

In a similar vein, Price et al. discussed a case of GBM in the right frontal lobe that later recurred in the corpus callosum, which contains the highest density of white matter.^16^, ^46^ Following an initial excision of tumor, the patient underwent radiotherapy, with recurrence 9 months later revealed by CT imaging and MRI in the contralateral left frontal lobe, with no evidence of tumor in the corpus callosum on T1‐ and T2‐weighted imaging.^16^ DTI at that time, however, produced an FA map that showed abnormalities in the genu of the corpus callosum, with a decrease in FA in peritumoral areas; there were also abnormalities in the *p* and *q* maps, with low *q* values and increased *p* values in the higher slices of the corpus callosum.^16^ Similar signatures were found in the recurrent tumor.^16^ The patient underwent a second resection, but several weeks following the identification of the first recurrence on imaging that prompted a second operation, the patient became symptomatic again, and a CT scan revealed tumor in the corpus callosum in the same region where diffusion tensor abnormalities were discovered 6 weeks earlier.^16^ This is yet another example of the utility of DTI for detecting recurrence earlier than conventional imaging.

Likewise, Wei et al. reported two cases where distant GBM recurrence was undetected by preoperative T1 contrast (T1c) images.^17^ Wei et al. studied the structural connectome in patients with GBM, with white matter connection strengths derived by joining a white matter connection template with a skeletonized FA map (produced by combining diffusion MRIs with a tensor model using the FMRIB Software Library [v6.0, Oxford, UK, https://fsl.fmrib.ox.ac.uk/fsl]).^17^ Two patients showed a primary tumor on preoperative T1c images, with diffusely infiltrated white matter connections, whereas the recurrence for each patient was in a distant region not visible on preoperative T1c images but linked to the primary tumor via white matter connections.^17^ Jin et al., Price et al., and Wei et al. are 3 studies in particular that reported the detection of recurrence using DTI, or a derivative of a diffusion tensor model (ie, skeletonized FA maps in Wei et al.), before the recurrence was visible on conventional imaging.^15^, ^16^, ^17^ Similar to Wei et al., with focus on the use of connectomes to trace occult tumor invasion, Krishnan et al. reported diffusion paths from the site of the primary tumor to secondary sites of progression, with a diffusion path reconstructed from the primary tumor in 1 patient that predicted the site of progression.^38^ Wei et al. and Krishnan et al. provide evidence for the importance of connectomics and tractography for predicting tumor spread, both locally and distantly, while also reiterating that tumor cells migrate along white matter tracts.^17^, ^38^

In addition to Jin et al. and Price et al., 4 additional studies (Khayal et al., Metz et al., Stecco et al., and Yan et al.) reported lower FA values in regions of recurrence.^15^, ^16^, ^29^, ^30^, ^31^, ^32^ From a different angle, compared to radiation necrosis and pseudoprogression (treatment‐related changes, ie, gliosis and/or radiation‐induced reactions), 2 studies found the FA values of true progression to be higher.^33^, ^34^ The collective findings from these studies suggest that FA values are typically lower in regions of recurrence/progression but are higher in recurrence regions compared to areas of radiation necrosis/treatment‐induced changes. First, to explain the reduction of FA in the presence of tumor, there is less anisotropic water diffusion in the setting of disrupted axonal fibers and white matter tracts caused by such a lesion.^16^ In GBM patients, lower FA values have also been shown to be associated with a reduced fiber density index in peritumoral regions.^47^ FA is a measure of both the directionality and the integrity of white matter tracts, correlated with cell density and proliferation; lower FA values are therefore seen in regions with aggressive, devastating tumor invasion and growth.^48^ In a study that fell outside of the articles returned by our search, the median FA value in non‐enhancing peritumoral regions with later tumor recurrence was lower than in non‐enhancing regions without later tumor recurrence.^48^

Notably, our review findings also show that FA is even lower in the presence of radiation necrosis than it is in the presence of recurrent tumor, and this is explained by the fact that in necrotic areas, nearly all axonal fibers and cells are defaced, and diffusion directions are at a minimum.^49^ This has been clinically demonstrated by Xu et al., who conducted a study on glioma patients who underwent postoperative radiotherapy.^50^ These patients had a new CE lesion on conventional MRI at the site of a previously postoperative radiotherapy‐treated glioma.^50^ Compared to those patients in whom this new CE lesion was diagnosed as recurrent tumor, the FA was significantly lower in patients diagnosed instead with radiation injury at that site.^50^ Kashimura et al. similarly found lower FA values in enhancing areas in a case of radiation necrosis compared to two cases of recurrent tumor in glioma patients, following postoperative radiotherapy treatment.^51^ These findings corroborate the explanation in theory for a lower FA in the presence of radiation injury compared to recurrent tumor.

Additionally, compared to the FA metric, there was more variability in reported findings for *p* and *q* values in regions of recurrence. This can be explained by likely differences amongst regions of interests analyzed, or simply due to the complexity of *p* and *q* in tumor regions overall. DTI‐*p* is a representative metric of MD, with MD sensitive to the diffusion of water molecules and thereby decreased in the setting of diffusion restriction by increased tumor cellularity.^52^ The *p* value can be formulized as follows ^21^:

$$p=\sqrt{3}\left(\mathrm{MD}\right)$$

Yan et al. reported significantly decreased *p* values in regions of tumor progression versus non‐progression, and Metz et al. reported lower MD values in regions of recurrence.^30^, ^32^ Li et al. also reported that lower *p* values in non‐enhancing regions were positively and significantly associated with rate of tumor progression.^36^ In contrast, DTI‐*q* is a metric associated with FA, and it can be represented mathematically as follows^21^:

$$q=\sqrt{{\left(\lambda_{1}-\mathrm{MD}\right)}^{2}+{\left(\lambda_{2}-\mathrm{MD}\right)}^{2}+{\left(\lambda_{3}-\mathrm{MD}\right)}^{2}}$$

Notice similar terms in the formulae for *q* and FA. As several studies reported a lower FA in regions of recurrence, Price et al. also reported a low *q* in recurrent tumor.^16^ This can be explained by the disruption to white matter tracts, whereas both the integrity of the tracts and directionality along the tracts are encumbered upon by infiltrating tumor.^16^

Finally, Metz et al. and Peeken et al. demonstrated the improved utility of FWC FA in predicting recurrence.^30^, ^41^ Metz et al. reported that FWC‐FA maps show significant distinctions between regions of recurrence and those of recurrence‐free edema, whereas these differences are less pronounced with non‐corrected FA maps.^30^ Peeken et al. also demonstrated the benefit of FWC DTI scans, using an FWC‐FA map to define an infiltrative area of tumor that was shown to overlap with an area of GBM recurrence.^41^ This is likely due to the fact that correcting for free water minimizes the effects of both edema and CSF presence, making DTI analyses more specific for pathologic tissue damage.^53^

Overall, DTI is sensitive to microstructural changes in white matter and offers practical utility for applications in various pathologies, including (but not limited to) brain tumors, multiple sclerosis, amyotrophic lateral sclerosis, stroke, Parkinson's disease, traumatic brain injury, and Alzheimer's disease.^45^ However, there are several limitations to DTI and tractography analyses that preclude a robust integration of this modality into clinical workflows, principally the variability in how DTI data is acquired and processed.^54^ Another limitation from the standpoint of fiber tracking is that the diffusion tensor model assumes a Gaussian distribution of fibers per voxel: that is, each voxel contains one population of fiber.^55^ However, we know that this is not universally true, as complex microstructural environments, such as a tumor microenvironment, are non‐Gaussian, where several crossing fibers and complex architecture can exist per voxel.^55^

With respect to fiber tracking, there are two main categories of tractography: deterministic and probabilistic. In deterministic streamline tractography, tracts are seeded at one point and grown along the local vectors in a stepwise fashion; a streamline is stopped when it reaches a point of high uncertainty.^56^ This high uncertainty can be thresholded based upon FA values, and when a streamline encounters a point that falls below a certain FA value, it stops in order to avoid mounting errors with each next step in building the streamline trajectory.^56^ Notably, areas with low FA values typically correlate with areas of large uncertainty with respect to the principal diffusion direction.^56^ As we found in this systematic review, areas of tumor recurrence have relatively low FA values. With tumor areas having relatively low anisotropy values in general, deterministic tractography becomes suboptimal and unreliable in the presence of such pathology.^57^

One possible mitigating mechanim, however, is the use of streamline atlases. In the literature, for example, Salvalaggio et al. used an average streamline map constructed from a Human Connectome Project (HCP) atlas, containing average white matter streamline counts per voxel within the HCP template; they then overlaid patient GBM tumor masks onto this map and calculated the average streamline count per voxel in the tumor area, which they termed a “tract density index.”^58^ Salvalaggio et al. showed that in patients with GBM, overall survival was prolonged when GBM populates areas with a low tract density index, and this relationship was found to be significant.^58^ This is particularly intriguing, in light of both contemporary and historical evidence (dating back to Scherer in the 1930s and 1940s) for the infiltration of white matter by GBM and other gliomas, as well as the growth of GBM alongside white matter tracts.^20^, ^59^, ^60^, ^61^, ^62^, ^63^, ^64^, ^65^, ^66^, ^67^, ^68^ Given the evidence for white matter tract invasion by GBM, Salvalaggio et al.’s finding that GBM growth in areas with a lower tract density index correlates with a more favorable prognosis is especially interesting.^58^

Furthermore, probabilistic tractography overcomes some of the limitations of deterministic tractography; it is intended for handling areas of high uncertainty and representing that uncertainty by quantifying the confidence of a streamline trajectory from one region to any number of endpoint regions.^56^ Barajas et al. used probabilistic streamline tractography to generate track density maps for patients with GBM, finding that in areas with “aggressive histopathologic features,” there was an increased likelihood of higher track density, whereas “relative track density” was significantly associated with architectural disturbance.^69^ Additionally, Kis et al. used probabilistic tractography in high‐grade glioma patients (including GBM patients) to investigate the breadth of tumor infiltration at diagnosis; they found that the results of probabilistic tractography, when thresholded at 5% and overlapped with the recurrence region, had a sensitivity and specificity of 81% and 90%, respectively, for predicting the initial breadth of tumor.^57^

Overall, probabilistic tractography is a prudent choice for complex environments, such as brain tumors with aggressive histopathology or regions with crossing fibers and not one single fiber population.^57^ Although probabilistic tractography algorithms are not without their own limitations in the presence of tumor, as is the case with deterministic tractography, these fiber‐tracking techniques are a promising mechanism for investigating disrupted tracts in the presence of infiltrating pathology, and they can provide us with prognostic foresight into clinical outcomes.^57^ The utility of tractography invites additional exploration of how these algorithms can be improved and used for predicting tumor progression and guiding treatment.

Finally, and despite certain limitations, there is ample literature reporting the clinical value and potential of DTI overall, as supported here and in a multitude of other studies and systematic reviews. Brancato et al., for example, conducted a systematic review of DTI metrics used to predict survival in patients with GBM, where metrics such as MD, FA, and *q* were shown to be useful for predicting overall and/or progression‐free survival.^25^ In this present study, however, we instead focused on the specific question of how DTI can be useful for predicting progression in patients with GBM. Although a minority of GBM patients have leptomeningeal dissemination and distant progression (5.3% and 6.1% of 247 isocitrate dehydrogenase‐wildtype GBM patients, respectively, reported in a study by Jiang et al.) compared to local progression (75.3% in the same study), wherein leptomeningeal progression may limit the utility of DTI given that white matter does not reside there, most patients do progress locally.^10^ Overall, having insight into the local and even distant white matter infiltrative trajectory that this devastating tumor will take may better inform and guide the treatment regimen. With that, a remote objective for DTI use is its standardized integration into surgical and radiotherapy protocols and planning, wherein training in DTI acquisition, processing, and interpretation may make that a more feasible endeavor and prove instrumental for clinicians in the field. In order to facilitate this goal, however, a refinement and robust validation of DTI acquisition, processing methodologies, analysis, and interpretation are essential, so that a more ubiquitous understanding of its clinical applicability is achieved for systematic integration into practice.

### Limitations

The primary limitation of this systematic review is the overall poor quality of articles included, all of which had a level of evidence of 4. The included articles were limited themselves, predominantly, by small sample sizes, single‐center patient populations, retrospective designs, as well as time, cost, and technical constraints. Furthermore, some of the included studies did not provide extensive details about the treatment administered pre‐recurrence, and so we could only include the information that was reported. Moreover, the utility of DTI for predicting/characterizing recurrence was made in comparison to the standard imaging modality for diagnosing GBM and monitoring GBM response to treatment, which is conventional MR imaging. Discussion of other advanced imaging techniques was not included within the scope of this review.

Additionally, although we could opine on the utility of DTI for earlier detection of recurrence/progression of GBM compared to conventional imaging, we could not comment on the precise time interval between first treatment and the DTI study that detected recurrence/progression for all studies, as this information was not reliably available amongst all included articles. Moreover, a standard definition of recurrence/progression did not exist across the 16 included articles, whereas some studies defined progression according to changes on surveillance MR imaging, histopathological confirmation during a second resection, Macdonald criteria, clinico‐radiological changes assessment and treatment response monitoring as per RANO criteria, or symptomatic recurrence imaging. This serves as another limitation with respect to variation in how recurrence/progression may have been defined, reported, and assessed across studies.

## CONCLUSIONS

GBM recurrence is an inevitable reality for many patients, irrespective of multimodal treatment. DTI has been shown to detect and predict recurrence pathways prior to recurrent tumor becoming visible on conventional imaging, with certain patterns that can be predictive of progression. This systematic review unveiled several common trends throughout the literature pertaining to DTI metrics and associated tractography/connectomics. This valuable information may help guide both surgical and radiotherapy planning with the aim of prolonging progression‐free and overall survival. Of particular interest is the use of DTI in predicting and distinguishing local versus distant tumor progression, which may offer additional insight into a patient's individual and heterogeneous GBM profile. The increasing evidence in the literature of DTI's utility for diagnostic and prognostic purposes is a call to consider its standardized implementation into clinical workflows and training in the future.

## CONFLICT OF INTEREST STATEMENT

The authors declare no conflicts of interest.
